# Supplementary material for: Environmental and Occupational Short-Term Exposure to Airborne Particles and FEV1 and FVC in Healthy Adults: A Systematic Review and Meta-Analysis
Source: Int J Environ Res Public Health. 2021 Oct 9;18(20):10571. doi: 10.3390/ijerph182010571 (PMC8536058; doi:10.3390/ijerph182010571)
Supplement: Supplementary file 1 [file ijerph-18-10571-s001.zip › ijerph-1367551-supplementary.pdf]

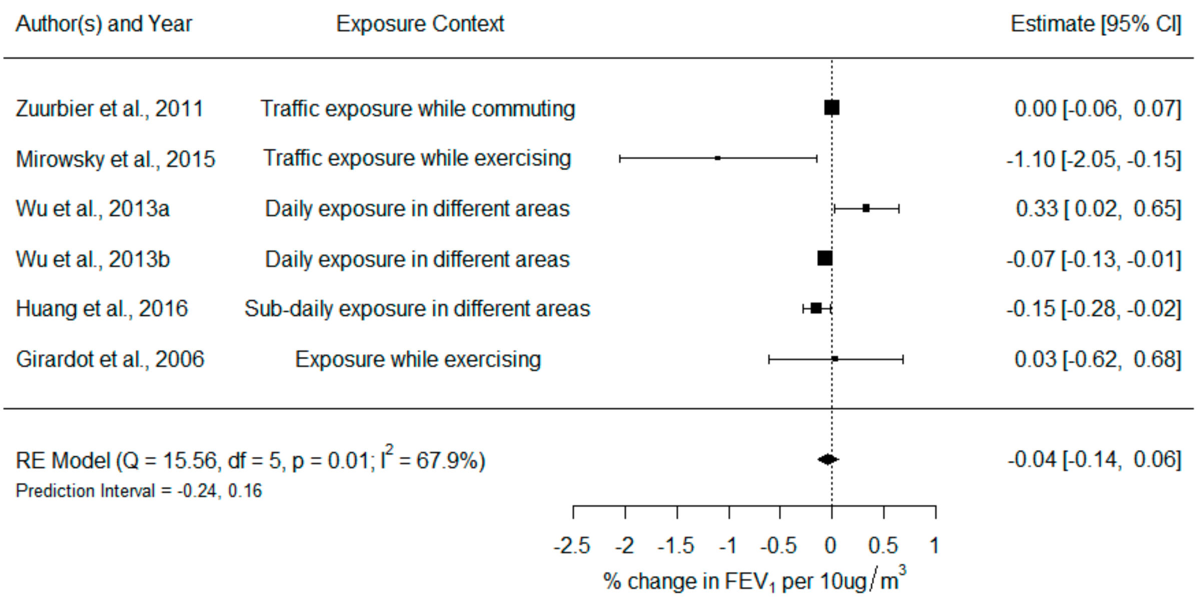

Figure S1 - Meta-analyses of the association between environmental $PM_{2.5}$ and FEV_1 (% change). Random-effect meta-estimate is indicated by vertical point of diamond and 95% CI is represented by horizontal point. Squares represent individual effect size of primary studies and the bars the 95% CI; size of squares is proportional to weight in calculating random-effect summary estimates.

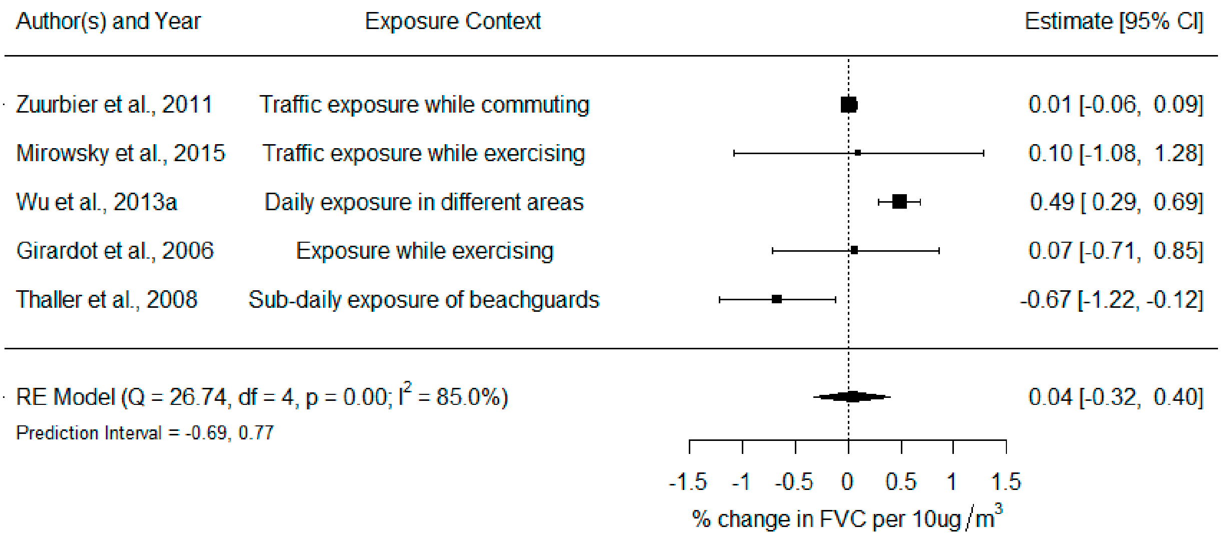

Figure S2 - Meta-analyses of the association between environmental $PM_{2.5}$ and FVC (% change). Random-effect meta-estimate of association is indicated by vertical point of diamond and 95% CI is represented by horizontal point. Squares represent individual effect size of primary studies and the bars the 95% CI; size of squares is proportional to weight in calculating random-effect summary estimates.

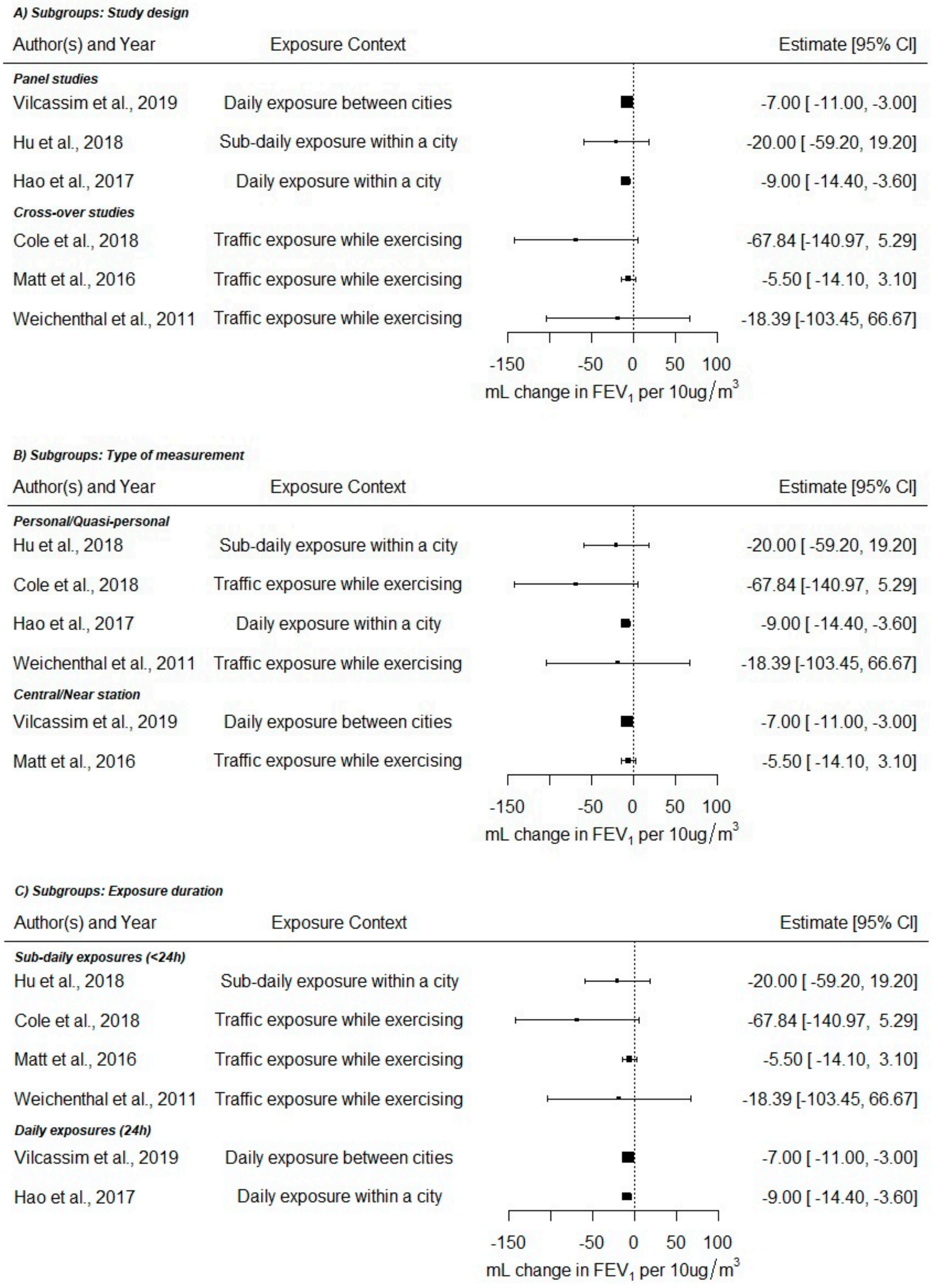

Figure S3 - Forest plot of the association between environmental $PM_{2.5}$ and FEV_1 (mL change) grouped by (A) study design, (B) type of measurement and (C) exposure duration. Squares represent individual effect size of primary studies and the bars the 95% CI.

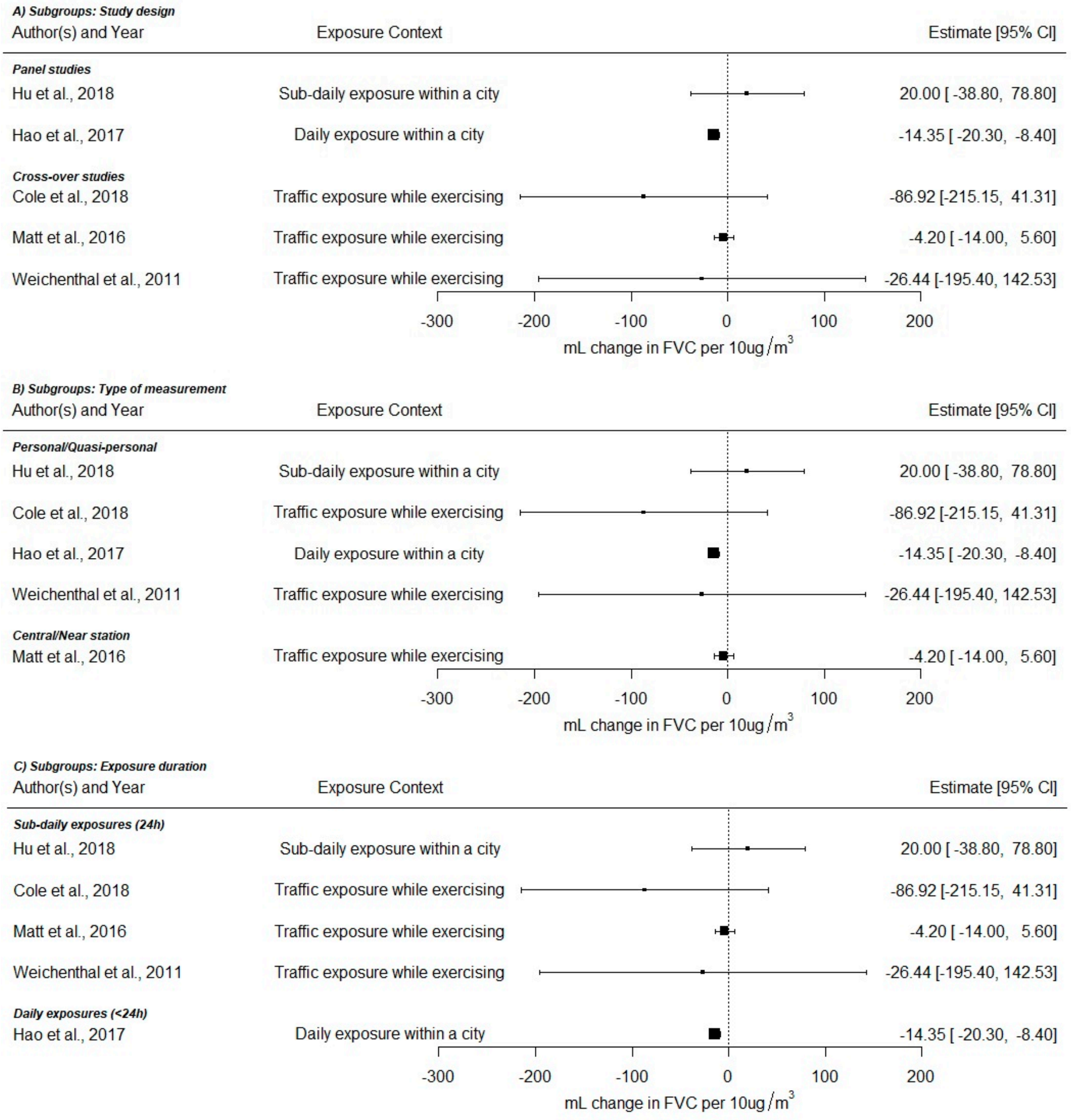

Figure S4 - Forest plot of the association between environmental $PM_{2.5}$ and FVC (mL change) grouped by (A) study design, (B) type of measurement and (C) exposure duration. Squares represent individual effect size of primary studies and the bars the 95% CI.

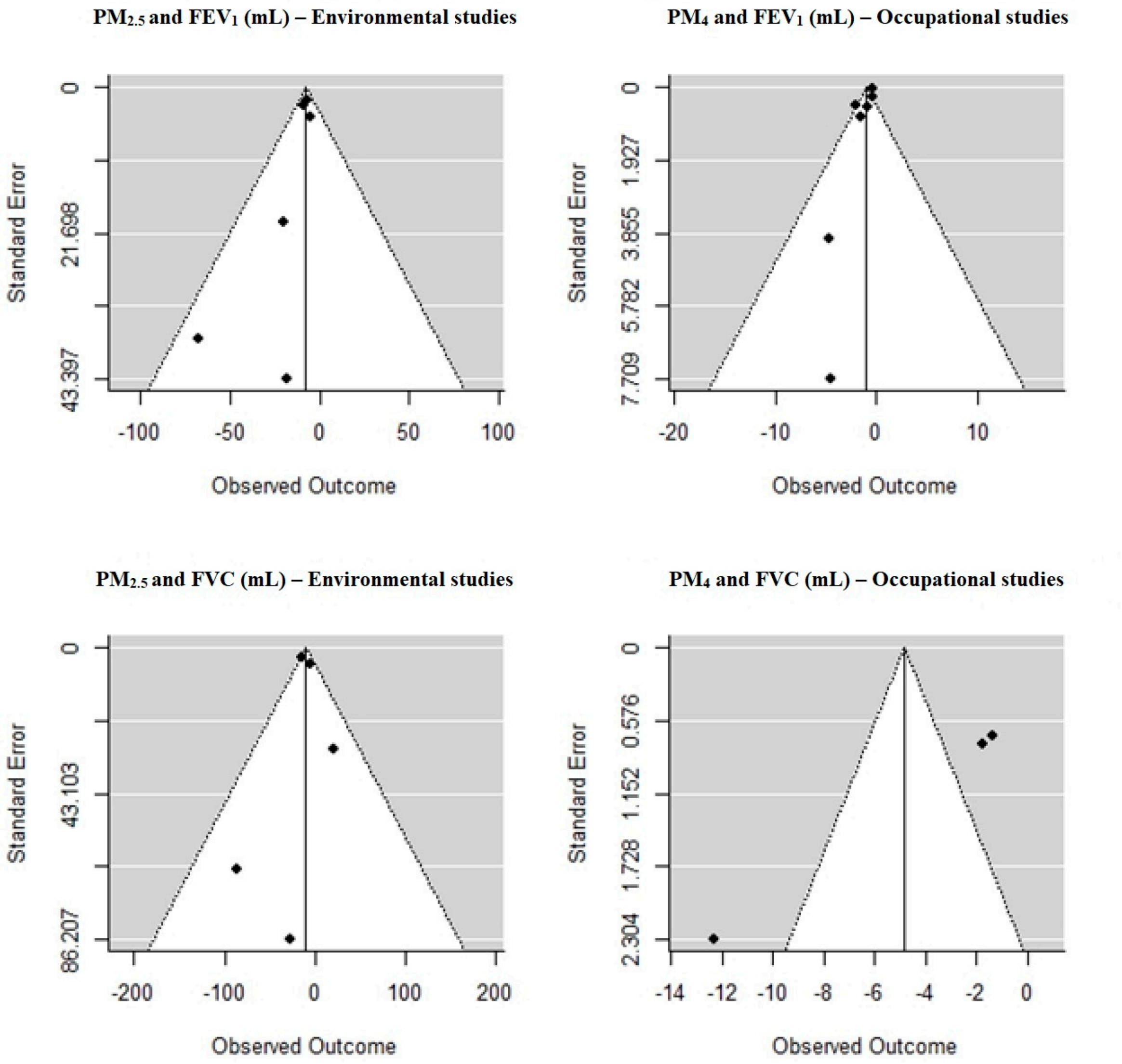

Figure S5 - Funnel plots of FEV_1 and FVC (mL change) meta-analyses for environmental and occupational studies.

Supplementary file 1 - Search Strategy and Keywords

#1 TOPIC: ("Lung Inflammation*") OR TOPIC: ("Pulmonary Inflammation*") OR TOPIC: ("Respiratory Inflammation*") OR TOPIC: ("Exhaled nitric oxide") OR TOPIC: (FeNO) OR TOPIC: (eNO)

#2 TOPIC: ("Lung Function*") OR TOPIC: (Spirometry) OR TOPIC: ("Pulmonary Function*") OR TOPIC: ("Airway Function*") OR TOPIC: ("Forced Expiratory Volume") OR TOPIC: ("Respiratory function*")

#3 TOPIC: ("ultrafine particle*") OR TOPIC: ("Particulate matter*") OR TOPIC: (particulate*) OR TOPIC: (UFP) OR TOPIC: (particle*) OR TOPIC: ("diesel exhaust*") OR TOPIC: (fume) OR TOPIC: (dust*) OR TOPIC: (PM2.5)

#4 #2 OR #1

#5 #4 AND #3

#6 TOPIC: (chronic) OR TOPIC: ("long term")

#7 TOPIC: (rat) OR TOPIC: (mice) OR TOPIC: (animal) OR TOPIC: (mouse)

#8 TOPIC: (cell) OR TOPIC: ("cell culture")

#9 TOPIC: (children)

#10 #5 NOT #6

#11 #10 NOT #7

#12 #11 NOT #8

#13 #12 NOT #9

Supplementary file 2- Additional FEV_1 results

FEV_1 (% change) and $PM_{2.5}$ in environmental studies

Figure S1 presents the association between environmental $PM_{2.5}$ exposures and FEV_1 measured in % change from a baseline value. Six studies were evaluated, and a negative but not significant association was found: -0.04% (95% CI: -0.14 to 0.06%; I^2 = 68%). The exclusion of Wu et al. (2013a) in the leave-one-out test resulted in a reduction in the value of the estimate: -0.07% (95% CI: -0.15 to 0.02%; I^2 = 60%).

Studies that could not be pooled in the meta-analyses

Seven environmental and six occupational studies could not be pooled in the meta-analyses because their outcomes (i.e. log % change from predicted value, log % change and % change from predicted value) or exposures metrics (i.e. log-transformed exposure, no information about IQR) were not comparable and could not be combined with at least three studies. Estimates and confidence intervals of these studies are presented in Table S2. Among the environmental studies, Dales et al. (2013) and Cakmak et al. (2014) reported negative association, Jarjour et al. (2013); Kubesch et al. (2015) and Mirabelli et al. (2015) reported a negative but non-statistically significant relationship and Baccarelli et al. (2014) and Liu et al. (2018) found a positive but non-statistically significant association between FEV_1 and fine particles. In occupational studies using a cross-shift design, Mandryk et al. (1999), Mandryk et al. (2000) and Neghab et al. (2018) found a statistically significant negative association in workers occupationally exposed to the respirable fraction of wood dust, while Hu et al. (2006) and Mitchell et al. (2015) reported a negative non-statistically significant exposure-outcome relationship.

Additional FVC results

FVC (% change) and $PM_{2.5}$ in environmental studies

No relationship was found for the % change in FVC after exposure to $PM_{2.5}$ in five environmental studies: 0.04% (95% CI: -0.32 to 0.40; I^2: 85%) (Figure S2). The exclusion of Wu et al. (2013a) caused a reduction in heterogeneity and resulted in a negative but not statistically significant association: -0.14% (95% CI: -0.51 to 0.24%; I^2: 49%).

Studies that could not be pooled in the meta-analyses

Five environmental and five occupational studies could not be pooled in the meta-analysis because their outcomes (i.e. log % change and % change from log-predicted value) or exposures metrics (i.e. log-transformed exposure, no information about IQR) were not comparable with other researches and could not be combined with at least three studies. In the environmental studies, Cakmak et al. (2014); Dales et al. (2013) and Jarjour et al. (2013) reported a negative but non-statistically significant association, while Baccarelli et al. (2014) and Kubesch et al. (2015) found a positive but non-statistically relationship between fine particles and FVC. In the occupational studies with a cross-shift design, Mandryk et al. (1999), Mandryk et al. (2000) and Neghab et al. (2018) found a statistically significant negative association between FVC and exposure to the respirable fraction of wood dust, while Hu et al. (2006) and Mitchell et al. (2015) did not report a statistically significant exposure-response relationship (Table S2).

Table S1 - PRISMA Checklist

Section/topic	#	Checklist item	Reported on page #
**TITLE**			
Title	1	Identify the report as a systematic review, meta-analy	1
**ABSTRACT**			
Structured summary	2	Provide a structured summary including, as applicable: background; objectives; data sources; study eligibility criteria, participants, and interventions; study appraisal and synthesis methods; results; limitations; conclusions and implications of key findings; systematic review registration number.	2
**INTRODUCTION**			
Rationale	3	Describe the rationale for the review in the context of what is already known.	3
Objectives	4	Provide an explicit statement of questions being addressed with reference to participants, interventions, comparisons, outcomes, and study design (PICOS).	4
**METHODS**			
Protocol and registration	5	Indicate if a review protocol exists, if and where it can be accessed (e.g., Web address), and, if available, provide registration information including registration number.	4
Eligibility criteria	6	Specify study characteristics (e.g., PICOS, length of follow-up) and report characteristics (e.g., years considered, language, publication status) used as criteria for eligibility, giving rationale.	4 and 5
Information sources	7	Describe all information sources (e.g., databases with dates of coverage, contact with study authors to identify additional studies) in the search and date last searched.	4
Search	8	Present full electronic search strategy for at least one database, including any limits used, such that it could be repeated.	4

Study selection	9	State the process for selecting studies (i.e., screening, eligibility, included in systematic review, and, if applicable, included in the meta-analysis).	4 and 5
Data collection process	10	Describe method of data extraction from reports (e.g., piloted forms, independently, in duplicate) and any processes for obtaining and confirming data from investigators.	6
Data items	11	List and define all variables for which data were sought (e.g., PICOS, funding sources) and any assumptions and simplifications made.	6
Risk of bias in individual studies	12	Describe methods used for assessing risk of bias of individual studies (including specification of whether this was done at the study or outcome level), and how this information is to be used in any data synthesis.	6
Summary measures	13	State the principal summary measures (e.g., risk ratio, difference in means).	6 and 7
Synthesis of results	14	Describe the methods of handling data and combining results of studies, if done, including measures of consistency (e.g., I^2) for each meta-analysis.	6 and 7
**Section/topic**	**#**	**Checklist item**	**Reported on page #**
Risk of bias across studies	15	Specify any assessment of risk of bias that may affect the cumulative evidence (e.g., publication bias, selective reporting within studies).	6
Additional analyses	16	Describe methods of additional analyses (e.g., sensitivity or subgroup analyses, meta-regression), if done, indicating which were pre-specified.	7 and 8
**RESULTS**			
Study selection	17	Give numbers of studies screened, assessed for eligibility, and included in the review, with reasons for exclusions at each stage, ideally with a flow diagram.	8 and Figure 1
Study characteristics	18	For each study, present characteristics for which data were extracted (e.g., study size, PICOS, follow-up period) and provide the citations.	8 and 9, Table 1 and Table S2

Risk of bias within studies	19	Present data on risk of bias of each study and, if available, any outcome level assessment (see item 12).	9 and 10
Results of individual studies	20	For all outcomes considered (benefits or harms), present, for each study: (a) simple summary data for each intervention group (b) effect estimates and confidence intervals, ideally with a forest plot.	Table S2 and Figures 2 to 5
Synthesis of results	21	Present results of each meta-analysis done, including confidence intervals and measures of consistency.	10, 11 and 12 Figures 2 to 5
Risk of bias across studies	22	Present results of any assessment of risk of bias across studies (see Item 15).	12
Additional analysis	23	Give results of additional analyses, if done (e.g., sensitivity or subgroup analyses, meta-regression [see Item 16]).	10, 11 and 12
**DISCUSSION**			
Summary of evidence	24	Summarize the main findings including the strength of evidence for each main outcome; consider their relevance to key groups (e.g., healthcare providers, users, and policy makers).	12
Limitations	25	Discuss limitations at study and outcome level (e.g., risk of bias), and at review-level (e.g., incomplete retrieval of identified research, reporting bias).	15
Conclusions	26	Provide a general interpretation of the results in the context of other evidence, and implications for future research.	16
**FUNDING**			
Funding	27	Describe sources of funding for the systematic review and other support (e.g., supply of data); role of funders for the systematic review.	17

From: Moher D, Liberati A, Tetzlaff J, Altman DG, The PRISMA Group (2009). Preferred Reporting Items for Systematic Reviews and Meta-Analyses: The PRISMA Statement. PLoS Med 6(7): e1000097. doi:10.1371/journal.pmed1000097 .

Table S2 - Metrics of respiratory outcomes used across the studies

	Outcome Unit	Example of Codification	Definition
**Absolute change**	Absolute change	mL change	The difference between the value post-exposure and the value pre-exposure is calculated and included in the model
**Percent change**	Percent change (not log-transformed model)	% change	The difference between the post-exposure value and the pre-exposure (or mean) value is calculated, transformed in % change and then included in the model
	Percent change (log-transformed model)	log % change	The outcome is first log-transformed and then the difference post – pre exposure is calculated, transformed in % change and then included in the model
	Percent change from predicted value (PV)	%PV change	The absolute value of the outcome is transformed to % deviation from a mean predicted value from a reference population. The difference between post-exposure and the pre-exposure is calculated and then included in the model
	Percent change from PV (log-transformed model)	Log %PV change	The absolute value of the outcome is transformed to % deviation from a mean predicted value from a reference population. The difference between post-exposure and the pre-exposure is calculated, log-transformed and then included in the model

Table S3 - Descriptive results of the studies selected in the systematic review according to their outcomes.

Authors and Year	Pollutant	Statistical Approach	Outcome Unit	Outcome Change (SE or 95% CI or p- value)
**Forced Expiratory Volume in 1 Second (FEV_1)**				
Baccarelli et al. (2014)	$PM_{2.5}$	Change per 83.9 µg/m^3	log % change	0h: 1.11% (-1.31 to 3.59)
Cakmak et al. (2014)	$PM_{2.5}$	Change per 9 µg/m^3	log % PV	Lag 1: -0.42% (-0.83 to -0.004)
Cole et al. (2018)	$PM_{2.5}$	Change per 4.7 µg/m^3	mL change	0h: -32mL (-66 to 3.0)
Dales et al. (2013)	$PM_{2.5}$	Change per 9 µg/m^3	log % PV	Lag 1: -0.42% (-0.83 to -0.004)
Girardot et al. (2006)	$PM_{2.5}$	Change per 1 µg/m^3	% change	0h: 0.003% (0.033)
Hao et al. (2017)	$PM_{2.5}$	Change per 10 µg/m^3	mL change	Lag 0: -9mL (-14 to -3.6) // Lag 0-1: -1.7mL (-5.9 to 2.4)
Hu et al. (2018)	$PM_{2.5}$	Change per 10 µg/m^3	mL change	Lag 1: -20 mL (20)
Huang et al. (2016)	$PM_{2.5}$	Change per 10 µg/m^3	% change	During exposure: -0.13% (-0.24% to -0.05%) 0h: -0.15% (-0.3 to -0.02) // 3h: 0.19% (-0.02 to 0.38%) 5h: 0.14%(-0.09 to 0.35%) // 7h: 0.04%(-0.16 to 023%) 20h: 0.01% (-0.35 to 0.35%)
Jarjour et al. (2013)	$PM_{2.5}$	Change compared to baseline (T-test)	mL change	Low Traffic-0h: 20mL (p>0.05) // 4h: 40mL (p>0.05) High traffic-0h: 50mL (p>0.05) // 4h: -10mL (p>0.05)
Kubesch et al. (2015)	$PM_{2.5}$	IQR not informed	mL change	Pooled analysis for 30 min, 3h and 6h: -2mL (-23 to 18)
Liu et al. (2018)	$PM_{2.5}$	Change per 17.4 µg/m^3	% PV	0.3% (-5.1 to 5.7)
Matt et al. (2016)	$PM_{2.5}$	Change per 1 µg/m^3	mL change	0h: -0.55mL (-1.4 to 0.3) // 7h: 0.43mL (-0.5 to 1.4)
Mirabelli et al. (2015)	$PM_{2.5}$	Change per 20.9 µg/m^3	% PV	Non-asthmatics: 0h: −0.42% (−2.2, 1.3)
Mirowsky et al. (2015)	$PM_{2.5}$	Change per 1µg/m^3	% change	0h: -0.11% (-0.2 to -0.01) // 24h: -0.04% (-0.15 to 0.06)
Vilcassim et al. (2019)	$PM_{2.5}$	Change per 10 µg/m^3	mL Change	Evening: -7 mL (-11 to-3)
Weichenthal et al. (2011)	$PM_{2.5}$	Change per 8.7 µg/m^3	mL change	0h: -16mL (-90 to 58) // 1h: 32mL (-46 to 110) // 2h: 4.9 mL (-81 to 90) // 3h: 10mL (-50 to 69)
Wu et al. (2013a)	$PM_{2.5}$	Change per 51.2 µg/m3	% change	Lag 1: 1.7% (0.1 to 3.3)
Wu et al. (2013b)	$PM_{2.5}$	Change per 63.4 µg/m^3	% change	Morning: -0.5% (-1.0 to -0.07) // evening: -0.49% (-0.93 to -0.05)
Zuurbier et al. (2011)	$PM_{2.5}$	Changer per 68.1 µg/m3	% change	0h: 0.02% (-0.41 to 0.45) // 6h: 0.21% (-0.26 to 0.67)
Altin et al. (2002)	PM_4	Change compared to baseline (T-test)	mL change	Workers-0h: -102mL (p=0.56) Controls-0h: -60mL (p=0.56)
Bakirci et al. (2006)	PM_4	Change compared to baseline (Mann-Whitney)	mL change	Workers-0h: -120mL (-65 to -185) Controls-0h: 20mL (-65 to 105)
Bakirci et al. (2007)	PM_4	Change compared to baseline (T-test)	mL change	Cross-shift 1st day: -102mL (-137 to -67) Cross-shift 1st month: -78mL (-104 to -52) Cross-shift 3rd month: -50mL (-73 to -27)

				Cross-shift 6th month: -55mL (-85 to -25)<br>Cross-shift 12th month: -67mL (-100 to -34)
Fell et al. (2011)	PM_4	Change compared to baseline (T-test)	mL change	Non-asthmatics: 0h: -46 mL (-86 to 6.3)
Gaughan et al. (2014)	PM_4	Change compared to baseline (T-test)	mL change	0h: -45mL (25.7mL)
Herbert et al. (1994)	PM_4	Change compared to baseline (T-test)	mL change	0h: -39mL (p=0.044)
Hu et al. (2006)	$PM_{2.5}$	1% change in log dust	% PV	0h: -1.31% (0.85)
Mandryk et al. (1999)	PM_4	% change in log dust	% PV	0h after exposure: 6.3% reduction in workers compared to 1.78% reduction in controls (P<0.001)
Mandryk et al. (2000)	PM_4	% change in log dust	% PV	0h after exposure: 6.44% reduction for green mill 21.8% reduction in dry mill workers (P<0.001 compared to control)
Mitchell et al. (2015)	$PM_{2.5}$	1% change in log dust	mL change	0h: -0.05ml (-27.76 to 27.66)
Neghab et al. (2018)	PM_4	Cross-shift change. Exposed versus Controls	% PV	0h: -10.5% (-14.3 to -6.8)
Slaughter et al. (2004)	PM_4	Change per 1,000 µg/m^3	mL change	0h: -30mL (-87 to 26)
Ulfvarson and Alexandersson (1990)	PM_4	Cross-shift change. Exposed versus Controls	mL change	Workers-0h: -105.8mL (92mL)<br>Controls-0h: -44.8mL (45mL)
**Forced Vital Capacity (FVC)**				
Baccarell et al. (2014)	$PM_{2.5}$	Change per 83.9 µg/m^3	log % change	0h: 0.12% (-2.79 to 3.11)
Cakmak et al. (2014)	$PM_{2.5}$	Change per 9 µg/m^3	log % PV	Lag 1: -0.27% (-0.69 to 0.16)
Cole et al. (2018)	$PM_{2.5}$	Change per 4.7 µg/m^3	mL change	0h: -41mL (-102 to 19)
Dales et al. (2013)	$PM_{2.5}$	Change per 9 µg/m^3	log % PV	Lag 1: -0.41% (-0.88 to 0.05)
Girardot et al. (2006)	$PM_{2.5}$	Change per 1 µg/m^3	% change	0h: 0.007% (0.04)
Hao et al. (2017)	$PM_{2.5}$	Change per 10 µg/m^3	mL change	Lag 0: -14.3mL (-19.5 to -7.6) // Lag 0-1: -2.8mL (-12 to 0.39)
Hu et al. (2018)	$PM_{2.5}$	Change per 10 µg/m^3	mL change	Lag 1: 20mL (30)
Jarjour et al. (2013)	$PM_{2.5}$	Change compared to baseline (T-test)	mL change	Low Traffic-0h: -20mL (p>0.05) // 4h: -30mL (p>0.05) High traffic-0h: 0mL (p>0.05) // 4h: -50mL (p>0.05)
Kubesch et al. (2015)	$PM_{2.5}$	IQR not informed	mL change	Pooled analysis for 30 min, 3h and 6h: 14mL (-11 to 38)
Matt et al. (2016)	$PM_{2.5}$	Change per 1 µg/m^3	mL change	0h: -0.42mL (-1.4 to 0.56) // 7h: 0.38mL (-0.56 to 1.32)
Mirowsky et al. (2015)	$PM_{2.5}$	Change per 1µg/m^3	% change	0h: 0.01% (-0.1 to 0.13) // 24h: 0.05% (-0.07 to 0.17)
Wu et al. (2013a)	$PM_{2.5}$	Change per 51.2 µg/m3	% change	Lag 1: 2.5% (1.5 to 3.5)
Thaller et al. (2008)	$PM_{2.5}$	Change per 10 µg/m^3	% change	Non-asthmatics: 0h: -0.80% (-1.4 to -0.09)
Weichenthal et al. (2011)	$PM_{2.5}$	Change per 8.7 µg/m^3	mL change	0h: -23mL (-170 to 124) // 1h: 46mL (-84 to 175) // 2h: -17mL (-90 to 56) // 3h: 2.5mL (-75 to 79)

Zuurbier et al. (2011)	$PM_{2.5}$	Changer per 68.1 µg/m3	% change	0h: 0.10% (-0.40 to 0.61) // 6h: 0.396% (-0.13 to 0.84)
Fell et al. (2011)	PM_4	Change compared to baseline (T-test)	mL change	Non-asthmatics: 0h: -41mL (-80 to 23)
Herbert et al. (1994)	PM_4	Change compared to baseline (T-test)	mL change	0h: -47mL (p=0.022)
Hu et al. (2006)	$PM_{2.5}$	1% change in log dust	% PV	0h: -1.42% (0.76)
Mandryk et al. (1999)	PM_4	% change in log dust	% PV	0h after exposure: 4.3% reduction in workers compared to 2.1% reduction in controls (P<0.01)
Mandryk et al. (2000)	PM_4	% change in log dust	% PV	0h after exposure: 1.46% reduction for green mill and 4.54% reduction in dry mill workers (P<0.001)
Mitchell et al. (2015)	$PM_{2.5}$	1% change in log dust	mL change	0h: -6.78 ml (-39.6 to 26.1)
Neghab et al. (2018)	PM_4	Cross-shift change. Exposed versus Controls	% PV	0h: -10.38% (-14.67 to -6.09)
Ulfvarson et al. (1990)	PM_4	Cross-shift change. Exposed versus Controls	mL change	Workers-0h: -283ml (53mL)<br>Controls-0h: 55mL (110mL)<br>P=0.01

Abbreviations: % change: percent change from a baseline or mean value; log % change: percent change of a log-transformed outcome; % PV: percent change from a predicted value; log % PV: percent change from a log-transformed predicted value.

$PM_{2.5}$: Particulate matter with median diameter of less than 2.5 µm; PM_4: Particulate matter with median diameter of less than 4 µm; SE: Standard error; 95% CI: 95% confidence interval.

Table S4 - Quality assessment of the studies included in the meta-analyses.

Criteria	Low risk	Medium risk	High risk
**Exposure assessment:** List of major considerations: 1) Adequacy of the method of exposure assessment to detect individual exposures. Personal and quasi-personal measurements are preferred to central station measurements 2) Equipments and direct-reading instruments were well described in the methods and are suitable for the type of measurement aimed. - **Low risk:** There is high confidence that the exposure to particles is the true exposure. - **Medium risk:** There is incertitude if the exposure measured represents the true exposure to particles, or one of the listed considerations is not applied. - **High risk**: There is direct evidence of high risk of misclassification bias, or the two listed considerations are not applied	**Zuurbier et al. (2011); Gaughan et al. (2014); Weichenthal et al. (2011); Hao et al. (2017); Cole et al. (2018); Bakirci et al. (2007); Slaughter et al. (2004); Ulfvarson et al. (1990); Mirowsky et al. (2015); Huang et al. (2016); Hu et al. (2018); Mirabelli et al. (2015)**	Risk of exposure misclassification due to the use of respirators: **Fell et al. (2011)** Measurements were not personal: **Wu et al. (2013a); Matt et al. (2016); Herbert et al. (1994); Wu et al. (2013b)**; **Girardot et al. (2006)**; **Dales et al. (2013)**; **Thaller et al. (2008); Vilcassim et al. (2019)**	**Bakirci et al. (2006)** (exposure based on historical records). **Altin et al. (2002)** (measurements for 60 min, no information if exposure and health outcomes were measured concomitantly, no details about methodology)
**Outcome assessment: Outcome assessment methods lack accuracy**. List of major considerations: 1) Spirometry was performed by a trained technician. 2) Spirometry was performed according to an official guideline. **- Low risk:** We have confidence that the outcome assessment reflects the true value of the physiological outcome.	**Zuurbier et al. (2011); Wu et al. (2013a); Gaughan et al. (2014); Bakirici et al. (2006); Weichenthal et al. (2011); Matt et al. (2016); Hao et al. (2017); Bakirci et al. (2007); Slaughter et al. (2004); Huang et**	Tests were not performed by a trained person or there is no information about it: **Fell et al. (2011); Cole et al., 2018; Altin et al. (2002); Matt et al. (2016); Mirowsky et al. (2015); Vilcassim et al. (2019); Mirabelli**	No information if test was performed by a trained technician and if procedure followed an official guideline: **Ulfvarson et al. (1990); Herbert et al. (1994)**

- **Medium risk:** There is incertitude if the outcome assessment represents the true value of the physiological outcome measured, or one of the listed considerations is not applied.<br>-**High risk**: There is direct evidence of high risk of misclassification bias, or the two listed considerations are not applied.	**al. (2016); Girardot et al. (2006); Dales et al. (2013); Thaller et al. (2008); Wu et al. (2013b); Hu et al. (2018)**	**et al. (2015)**	
**Confounding bias: Study appropriately accounted for all important well studied potential confounders and modifiers in the design or in the statistical analysis: Important effect confounders and modifiers: individual variables (e.g. age, sex, BMI (or height and weight)), health status (asthma, COPD), smoking status, temperature.**<br>-**Low risk**: study accounted for all important categories of confounders and modifiers which were measured consistently.<br>-**Medium risk**: study accounted for some but not all of confounders and modifiers, and this may introduce bias.<br>- **High risk:** study did not account for potential confounders and modifiers OR were inappropriately measured	**Zuurbier et al. (2011); Matt et al. (2016); Hao et al. (2017); Huang et al. (2016); Wu et al. (2013b); Girardot et al. (2006); Weichenthal et al. (2011); Mirowsky et al. (2015); Dales et al. (2013); Herbert et al. (1994); Thaller et al. (2008); Hu et al. (2018)**	No adjustment for individual variable between subjects, although controlled for within subjects by design: **Wu et al. (2013a)**<br>Occupational studies where it is not possible to differentiate co-exposure as cause of the effects: **Bakirci et al. (2006); Altin et al. (2002) ; Ulfvarson et al. (1990); Gaughan et al. (2014), Fell et al. (2011); Bakirci et al. (2007)**<br>No adjustment for temperature: **Cole et al., 2018; Slaughter et al. (2004); Vilcassim et al. (2019); Mirabelli et al. (2015)**	
**Selection bias: Does the selection of participants into**	**Zuurbier et al. (2011); Wu et al.**	Only male subjects: **Bakirci**	

**the study was done in a manner that might introduce bias in the study?** **-Low risk:** The descriptions of the studied population were sufficiently detailed and the risk of selection bias was minimal. **-Medium risk:** The description of the population is not complete or there is a possibility that the selection of the population may introduce bias. However, there is insufficient information about population to permit a judgment of high risk of bias. **-High risk:** There were indications from descriptions of the population of high risk of bias.	**(2013a); Gaughan et al. (2014); Weichenthal et al. (2011); Matt et al. (2016); Hao et al. (2017); Cole et al. (2018); Slaughter et al. (2004); Altin et al. (2002); Mirowsky et al. (2015); Huang et al. (2016); Girardot et al. (2006); Dales et al. (2013); Thaller et al. (2008); Fell et al. 2011; Bakirci et al. (2007); Hu et al. (2018); Vilcassim et al. (2019); Mirabelli et al. (2015)**	**et al. (2006); Ulfvarson et al. (1990); Wu et al. (2013b)** Employment duration not described: **Slaughter et al. (2004); Bakirci et al. (2006); Herbert et al. (1994); Ulfvarson et al. (1990)**	
**Selective reporting: Selective reporting of outcomes or analyses.** **-Low risk:** all of the studies pre-specified outcomes and findings were reported in the article or supplementary material **-Medium risk**: there was insufficient information about selective outcome to judge for low risk, but indirect evidence that suggests study was free of selective report. **-High risk**: not all pre-specified outcomes and findings were reported, or at least one of the primary outcomes was assessed with other methods than the pre-specified one, or at least one of the reported outcomes was not pre-specified	**Zuurbier et al. (2011); Wu et al. (2013a); Weichenthal et al. (2011); Matt et al. (2016); Hao et al. (2017); Cole et al. (2018); Bakirci et al. (2007); Slaughter et al. (2004); Altin et al. (2002); Herbert et al. (1994); Ulfvarson et al. (1990); Mirowsky et al. (2015); Wu et al. (2013b); Girardot et al. (2006); Dales et al. (2013); Thaller et al. (2008); Bakirci et al. (2006);**		Regression models described in the methods but results not presented: **Gaughan et al. (2014); Fell et al. (2011)**

	**Huang et al. (2016); Hu et al. (2018); Vilcassim et al. (2019); Mirabelli et al. (2015)**		
**Conflict of interest: Potential source of bias in reporting through source of funding** **-Low risk:** the study did not receive funding from an entity with financial interest in the outcome of study **-Medium risk**: there is insufficient information to judge for low risk, but indirect evidence suggests study was free of financial interest **-High risk**: study received support from an entity with financial interest in the outcome of study	**Zuurbier et al. (2011); Wu et al. (2013a); Gaughan et al. (2014); Bakirci et al. (2006); Weichenthal et al. (2011); Matt et al. (2016); Hao et al. (2017); Cole et al. (2018); Bakirci et al. (2007); Slaughter et al. (2004); Herbert et al. (1994); Mirowsky et al. (2015); Huang et al. (2016); Wu et al. (2013b); Girardot et al. (2006); Dales et al. (2013); Thaller et al. (2008); Hu et al. (2018); Vilcassim et al. (2019); Mirabelli et al. (2015)**	No information: **Altin et al. (2002); Ulfvarson et al. (1990)**	**Fell et al. (2011):** Funding from a possible interested organization (The European Cement Association)
**Incomplete outcome data: Was incomplete data adequately addressed?** **-Low risk:** no missing outcome data or missing data is unrelated to true outcome **-Medium risk:** there was insufficient information about incomplete data to judge for low risk, but indirect evidence	**Zuurbier et al. (2011); Wu et al. (2013a); Gaughan et al. (2014); Bakirci et al. (2006); Fell et al. (2011); Weichenthal et al. (2011); Matt et al. (2016); Hao et al.**		

suggests that incomplete data may introduce bias. **-High risk**: missing outcome data is related to true outcome	**(2017); Cole et al. (2018); Barkirci et al., 2007; Slaughter et al. (2004); Altin et al. (2002); Herbert et al. (1994); Ulfvarson et al. (1990); Mirowsky et al. (2015); Huang et al. (2016); Wu et al. (2013b); Girardot et., 2006; Dales et al. (2013); Thaller et al. (2008); Hu et al. (2018); Vilcassim et al. (2019); Mirabelli et al. (2015)**		
